# A Variational Level Set Approach Based on Local Entropy for Image Segmentation and Bias Field Correction

**DOI:** 10.1155/2017/9174275

**Published:** 2017-11-27

**Authors:** Jian Tang, Xiaoliang Jiang

**Affiliations:** ^1^College of Mechanical Engineering, Quzhou University, Quzhou, Zhejiang 324000, China; ^2^College of Mechanical Engineering, Southwest Jiaotong University, Chengdu, Sichuan 610031, China

## Abstract

Image segmentation has always been a considerable challenge in image analysis and understanding due to the intensity inhomogeneity, which is also commonly known as bias field. In this paper, we present a novel region-based approach based on local entropy for segmenting images and estimating the bias field simultaneously. Firstly, a local Gaussian distribution fitting (LGDF) energy function is defined as a weighted energy integral, where the weight is local entropy derived from a grey level distribution of local image. The means of this objective function have a multiplicative factor that estimates the bias field in the transformed domain. Then, the bias field prior is fully used. Therefore, our model can estimate the bias field more accurately. Finally, minimization of this energy function with a level set regularization term, image segmentation, and bias field estimation can be achieved. Experiments on images of various modalities demonstrated the superior performance of the proposed method when compared with other state-of-the-art approaches.

## 1. Introduction

Image segmentation has always been a crucial step in image understanding and computer vision. However, due to the limitations in imaging instrument and other external effects, bias field is often seen in many medical images which can be ascribed to a spatially varying field. Therefore, it is often a fundamental step to correct the bias field before performing quantitative analysis of the image data. Methods of bias field correction can be divided into two groups: prospective methods [1, 2] and retrospective methods [3, 4]. Prospective methods aim to avoid intensity inhomogeneity by using the shim techniques, special imaging sequences, or special hardware. However, the retrospective methods rely exclusively on the information of the acquired images and thus can extract information on intensity inhomogeneity.

Among all kinds of bias field correction methods, those based on segmentation are the most popular. In these methods, the tasks of segmentation and bias field correction are interleaved in an iterative process, thereby allowing their optimal results to be simultaneously achieved. In [5], Wells III et al. proposed an expectation-maximization (EM) algorithm for image segmentation and bias field estimation. However, such algorithm is sensitive to the choices of initial conditions, which limits its applications in automatic segmentation. Based on the EM algorithm, Meena and Shantha [6] presented a fully automated method for MR brain image segmentation by introducing the Fuzzy C-means (FCM) with spatial information. Also, a method of pixel relabeling is included to improve the segmentation accuracy. So, this method can very well be extended to segmentation of clinical MR brain images and the identification of pathologies. In [7], Xie et al. propose an approach joining a modified MRF classification and bias field estimation in an energy minimization framework, whose initial estimation is based on *k*-means algorithm in view of prior information on MRI. Thereby, this algorithm is also more reliable and effective.

Recently, the level set method has been applied to simultaneously segment images while estimating the bias field [8–15]. For example, Li et al. [8] introduced a local weighted *K*-means clustering-based variational level set approach to estimate the bias field and segment the images with intensity nonuniformity. A unique feature of this method is that the calculated bias field is essentially guaranteed by the data term in the variational formulation, without any extra effort to maintain the smoothness of the bias field. In [9], Zhang et al. presented a statistical and variational multiphase level set (SVMLS). This approach used the Gaussian distribution with spatially varying mean and variance to describe the image model. Thereby, it can distinguish regions with similar intensity means but different variances. In [10], Wang and Pan proposed a new image-guided regularization to restrict the level set function. In this method, tissue segmentation and bias field estimation are unified into a single Bayesian inference framework and are simultaneously achieved by minimizing the objective energy functional. So the method can be used for accurate segmentation and bias correction of medical images in the presence of severe intensity inhomogeneity. However, all of them are sensitive to initialization of the contour to some extent.

In this study, we propose a novel LGDF model in terms of noise and robustness for simultaneous image segmentation and bias field estimation. Firstly, according to the observed signal model of the image with intensity inhomogeneity, the LGDF energy function that includes the local entropy is defined for driving the evolution contour of the level set toward the desired boundary. The means of this objective function have a multiplicative factor that estimates the bias field in the transformed domain. Furthermore, by incorporating the bias field prior into a variational level set formulation with a regularization term, our method can be used for segmentation and bias field correction. Finally, we have also made some comparisons with several state-of-the-art models to show the superiority of our method over the traditional local region-based methods.

The contributions of this paper may be summarized as follows:

1. Our model is built by simultaneous segmentation and bias field correction within a single framework. Hence, it can make full use of a priori knowledge on the bias field and level set function.
2. By incorporating the local entropy information, our model can estimate the bias field more accurately.

## 2. Background

### 2.1. Li's Method

In order to overcome the problem of intensity inhomogeneity, Li et al. [8] introduced a variational level set approach to estimate the bias field and segment the images with intensity nonuniformity. The method is based on the following model to describe an observed image:

(1) $$\begin{array}{c} I=bJ+n, \end{array}$$

where *I* is the measure image, *b* is the bias field, *J* is the true image, and *n* is the additive noise. The assumption on the true image *J* and the bias field *b* has the following properties: (i) the bias field *b* is slowly varying in the image domain and (ii) the true image intensities *J* are approximately a constant within each class of tissue; that is, *J*(*x*) ≈ *c*_*i*_ for *x* ∈ *Ω*_*i*_, with {*Ω*_*i*_}_*i*=1_^*N*^ being a partition of *Ω*.

According to the above assumption, this method applies a circular neighborhood with a small radius *ρ* centered at each point *x* in the image domain *Ω*, defined by *O*_*x*_ = {*y* : |*y* − *x*| ≤ *ρ*}. Then, the value *b*(*y*) for all *y* in the circular neighborhood *O*_*x*_ can be well approximated by *b*(*x*). Therefore, the intensities *b*(*y*)*J*(*y*) in each subregion *O*_*x*_∩*Ω*_*i*_ are approximately the constant *b*(*x*)*c*_*i*_. Considering that the intensities *I*(*y*) in the neighborhood *O*_*x*_ can be classified into *N* classes, the local clustering criterion can be described as

(2) $$\begin{array}{c} E_{x}=\overset{N}{\underset{i=1}{\sum }}\int_{O_{x}\cap \Omega_{i}}K\left(\;x-y\;\right){\left|\;I\left(\;y\;\right)-b\left(\;x\;\right)c_{i}\;\right|}^{2}dy, \end{array}$$

where {*Ω*_*i*_}_*i*=1_^*N*^ denotes a partition of the image domain *Ω*, *b*(*x*)*c*_*i*_ is the cluster center to be optimized, and *K*(*x* − *y*) is a nonnegative weighting function.

### 2.2. Zhang's Method

In [9], Zhang et al. presented a statistical and variational multiphase level set method. Assuming that the mean and variance of the local Gaussian distribution are spatially varying parameters, *u*_*i*_(*x*) is the local intensity in the partition *O*_*x*_∩*Ω*_*i*_, so *u*_*i*_(*x*) can be approximated by *b*(*x*)*c*_*i*_:

(3) uix=∑y∈Ox∩ΩiIyΩ≈∑y∈Ox∩ΩibyJyΩ≈∑y∈Ox∩ΩibxciΩ=bxci,

where |*Ω*| is the number of pixels in *O*_*x*_∩*Ω*_*i*_.

By using maximum a posteriori probability and Bayes' rule [9], the local Gaussian distribution fitting energy can be described as follows:

(4) $$\begin{array}{c} E_{x}=\overset{N}{\underset{i=1}{\sum }}\lambda_{i}\int_{O_{x}\cap \Omega_{i}}-K\left(\;x-y\;\right)\log p_{i,x}\left(\;I\left(\;y\;\right)\;\right)dy, \end{array}$$

where *λ*_*i*_ are the positive constants and *p*_*i*,*x*_(*I*(*y*)) is the probability density in region *O*_*x*_∩*Ω*_*i*_, which is defined as

(5) $$\begin{array}{c} p_{i,x}\left(\;I\left(\;y\;\right)\;\right)=\frac{1}{\sqrt{2\pi }\sigma_{i}\left(x\right)}\exp\left(\;-\frac{{\left(I\left(y\right)-b\left(x\right)c_{i}\right)}^{2}}{2\sigma_{i}{\left(x\right)}^{2}}\;\right). \end{array}$$

## 3. The Proposed Scheme

### 3.1. Local Entropy

The definition of entropy has first been introduced by Shannon [16] and has been further developed by the information theory community. As far as segmentation is concerned, a region may be characterized using the average amount of information, namely, the entropy, carried by the intensity of the region, or using joint entropy for features combination. The entropy of an image is expressed as follows [17]:

(6) $$\begin{array}{c} E\left(\;I\;\right)=-\overset{N}{\underset{i=1}{\sum }}P_{i}\log P_{i}, \end{array}$$

where *P*_*i*_ is the probability of the given images *I*.

In this study, we give the definition of the local entropy in a spatially continuous domain *Ω*_*x*_ ⊂ *Ω*; then the local entropy of the point *x* can be written as

(7) $$\begin{array}{c} E\left(\;x,\Omega_{x}\;\right)=-\frac{1}{\log\left|\Omega_{x}\right|}\int_{\Omega_{x}}P\left(\;y,\Omega_{x}\;\right)\log P\left(\;y,\Omega_{x}\;\right)dy, \end{array}$$

where *P*(*y*, *Ω*_*x*_) is the grey level distribution. It is given by

(8) $$\begin{array}{c} P\left(\;y,\Omega_{x}\;\right)=I\left(\;y\;\right)\int_{\Omega_{x}}I\left(\;z\;\right)dz,\;y\in \Omega_{x}. \end{array}$$

**Figure 1:**
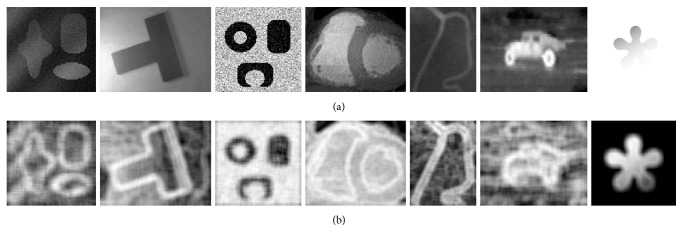
Results of the local entropy on images with noise and intensity inhomogeneity. (a) The original images. (b) The local entropy images.

We first apply (7) and (8) to compute the local entropy on images, which are displayed in Figure 1. As discussed in our previous work [18], the local entropy has good robustness for noise and intensity inhomogeneity.

### 3.2. New LGDF Energy Based on Local Entropy

As mentioned above, the energy *E*_*x*_ in (4) is sensitive to initialization and noise. In order to handle these problems, we use the local entropy *E*(*x*, *Ω*_*x*_) defined in (7) to describe the intensity variation in a neighbourhood *Ω*_*x*_ of a point *x*. Therefore, we redefined the new LGDF energy as follows:

(9) ExNLGDF=∑i=1NλiErx∫Ox∩Ωi−Kx−ylog⁡pi,xIydy,

where *E*_*r*_(*x*) = *E*(*x*, *B*(*x*, *r*)) is the local entropy of *x*, *B*(*x*, *r*) = {*y* : |*x* − *y*| ≤ *r*}, *r* > 0, and *K*(*x* − *y*) is a truncated Gaussian kernel, where *K*(*x* − *y*) = 0 for |*x* − *y*| ≥ *r*.

The ultimate goal is to minimize *E*_*x*_^NLGDF^ for all the center points *x* in the image domain *Ω*, which directs us to define the following energy:

(10) ENLGDF=∑i=1Nλi∫ΩErx·∫Ωi−Kx−ylog⁡pi,xIydydx.

Substituting (5) into (10), we can obtain the proposed energy formulation as follows:

(11) $$\begin{array}{c} E^{NLGDF}=\overset{N}{\underset{i=1}{\sum }}\lambda_{i}\int_{\Omega }E_{r}\left(\;x\;\right)\left(\;\int_{\Omega_{i}}K\left(\;x-y\;\right)\left(\;\log\left(\sqrt{2\pi }\sigma_{i}\left(x\right)\right)+\frac{{\left(I\left(y\right)-b\left(x\right)c_{i}\right)}^{2}}{2\sigma_{i}{\left(x\right)}^{2}}\;\right)dy\;\right)dx. \end{array}$$

### 3.3. Level Set Formulation

In this section, level set formulation is used to solve our energy *E*^NLGDF^ in (11). Let *ϕ* : *Ω* → *R* be a level set function; then its signs partition the domain into two disjoint regions *Ω*_1_ = {*x* : *ϕ*(*x*) > 0} and *Ω*_2_ = {*x* : *ϕ*(*x*) < 0}. We assume that the image domain *Ω* can be separated into two regions *Ω*_1_ and *Ω*_2_. These two regions can be represented by their membership functions defined as *M*_1_(*ϕ*(*x*)) = *H*(*ϕ*(*x*)) and *M*_2_(*ϕ*(*x*)) = 1 − *H*(*ϕ*(*x*)), respectively, where *H*(∙) is the Heaviside function. Thus, the energy in (11) can be written as the following level set formulation:

(12) $$\begin{array}{c} E^{NLGDF}\left(\;\phi ,c_{i},\sigma_{i},b\;\right)=\overset{2}{\underset{i=1}{\sum }}\lambda_{i}\int_{\Omega }E_{r}\left(\;x\;\right)\left(\;\int_{\Omega }K\left(\;x-y\;\right)\left(\;\log\left(\sqrt{2\pi }\sigma_{i}\left(x\right)\right)+\frac{{\left(I\left(y\right)-b\left(x\right)c_{i}\right)}^{2}}{2\sigma_{i}{\left(x\right)}^{2}}\;\right)M_{i}\left(\;\phi \left(y\right)\;\right)dy\;\right)dx. \end{array}$$

In our implementation, Heaviside function *H* is usually approximated by a smoothing function *H*_*ε*_ defined by

(13) $$\begin{array}{c} H_{\varepsilon }\left(\;x\;\right)=\frac{1}{2}\left[\;1+\frac{2}{\pi }\arctan\left(\;\frac{x}{\varepsilon }\;\right)\;\right]. \end{array}$$

The derivative of *H*_*ε*_ is the following smoothing function:

(14) $$\begin{array}{c} \delta_{\varepsilon }\left(\;x\;\right)=H_{\varepsilon }^{\prime }\left(\;x\;\right)=\frac{1}{\pi }\frac{\varepsilon }{\varepsilon^{2}+x^{2}}. \end{array}$$

To derive an accurate and smooth contour, we need to add a length term *L*(*ϕ*) and a regularization term *R*(*ϕ*). Therefore, the entire energy functional is

(15) Fϕ,ci,σi,b=ENLGDFϕ,ci,σi,b+νLϕ+μRϕ,

where

(16) Lϕ=∫∇Hϕxdx,Rϕ=∫12∇ϕx−12dx

and *ν* and *μ* are positive constants.

By minimization of the energy *F*(*ϕ*, *c*_*i*_, *σ*_*i*_, *b*) in (15), image segmentation and bias field estimation can be simultaneously achieved. The procedure is as follows: in each iteration, we minimize the energy *F*(*ϕ*, *c*_*i*_, *σ*_*i*_, *b*) with respect to each of its variables *ϕ*, *c*_*i*_, *σ*_*i*_, and *b*, given the other three updated in previous iteration. The energy minimization with respect to each variable can be obtained as follows.

For fixed *c*_*i*_, *σ*_*i*_, and *b*, minimization of the energy *F* in (15) with respect to *ϕ* can be obtained by solving the following gradient flow equation:

(17) $$\begin{array}{c} \frac{\partial \phi }{\partial t}=-\frac{\partial F}{\partial \phi }, \end{array}$$

where ∂*F*/∂*ϕ* is the Gâteaux derivative of the functional *F*.

The Gâteaux derivative can be obtained by using calculus of variation [19]; hence the gradient flow equation is expressed by

(18) ∂ϕ∂t=−δεϕλ1e1−λ2e2+νδεϕdiv∇ϕ∇ϕ+μΔϕ−div∇ϕ∇ϕ,

where *e*_1_ and *e*_2_ are the functions as follows:

(19) eix=∫ΩKx−yErx·log⁡2πσix+Iy−bxci22σix2dy.

For fixed *ϕ*, *σ*_*i*_, and *b*, the optimal *c*_*i*_ that minimizes the energy *F* is given by

(20) $$\begin{array}{c} c_{i}=\frac{\int \left(K*b\right)IM_{i}\left(\phi \left(x\right)\right)dx}{\int \left(K*b^{2}\right)M_{i}\left(\phi \left(x\right)\right)dx}. \end{array}$$

By fixing the other variables in (15), we obtain the minimizer of *b* as follows:

(21) $$\begin{array}{c} b=\frac{\sum_{i=1}^{2}K\left(x-y\right)\left(IM_{i}\left(\phi \left(x\right)\right)\right)\left(c_{i}/\sigma_{i}^{2}\right)}{\sum_{i=1}^{2}K\left(x-y\right)\left(M_{i}\left(\phi \left(x\right)\right)\right)\left(c_{i}/\sigma_{i}^{2}\right)}. \end{array}$$

By fixing the other variables in (15), we find an optimal *σ*_*i*_ as follows:

(22) σi2=∫∫Kx−yIy−cibx21−Miϕxdy dx∫∫Kx−y1−Miϕxdy dx.

*Remark*. The minimization problem in (18) is nonconvex, so we need to make the convergence criteria. The terminal condition is similar to |*F*^(*n*)^ − *F*^(*n* + 1)^| < 0.001 or the number of iterations that is set in advance. If the convergence criteria are reached, stop the iteration.

### 3.4. Description of Algorithm Steps

For a deep understanding of our model, the iterative procedure is summarized below in this section.

**Step 1 .**

Input the original image *I*(*x*).

**Step 2 .**

Compute the local entropy in (7) and (8).

**Step 3 .**

Initialize the level set function *ϕ* = *ϕ*^0^(*x*):

(23) $$\begin{array}{c} \phi^{0}\left(\;x\;\right)=\left\{\begin{array}{cc} -c_{0} & x\text{ is inside }C\\ 0 & x\in C\\ c_{0} & x\text{ is outside }C. \end{array}\right. \end{array}$$

**Step 4 .**

Initialize the parameters Δ*t*, *λ*_1_, *λ*_2_, *μ*, *σ*, and *ν*.

**Step 5 .**

Update *c*_*i*_, *b*, and *σ*_*i*_ by using (20), (21), and (22), respectively.

**Step 6 .**

Update the level set function *ϕ* according to (18).

**Step 7 .**

Check the convergence criteria and iteration number. If the iteration number reached a predetermined maximum number or |*F*^(*n*)^ − *F*^(*n* + 1)^| < 0.001 (*F*^(*n*)^ is the *n*th iteration result of *F*), stop the iteration; otherwise, return to Step 5.

## 4. Experimental Results

In this subsection, Li's model [8], Zhang's model [9], Wang's model [10], RSF model [20], LGDF model [21], and the method of this paper are applied on a variety of synthetic images and medical images. The algorithm is implemented in Matlab 2011a on a 2.8 GHz Intel Pentium IV personal computer. Unless otherwise specified, the parameters are set as follows: iteration time step Δ*t* = 0.1, weighting coefficients *λ*_1_ = *λ*_2_ = 1.0, *μ* = 1.0, and Gaussian kernel *σ* = 3.

### 4.1. Segmentation of Synthetic and Real Images

**Figure 2:**
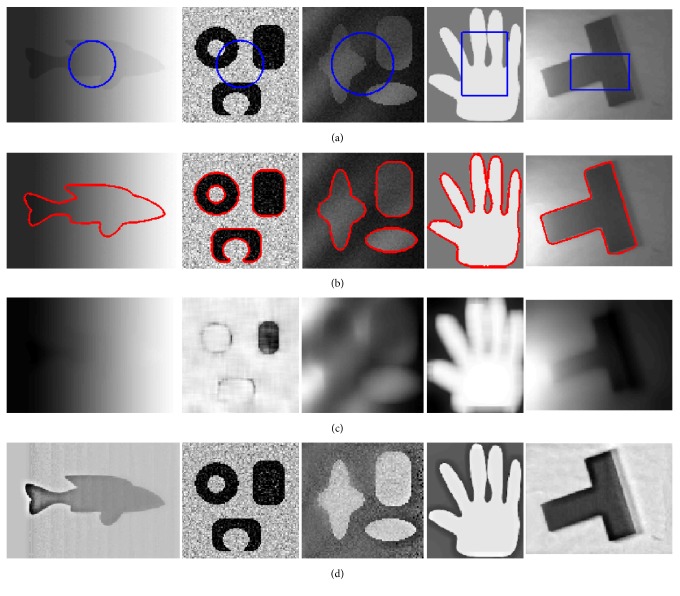
Application of our method to synthetic images. (a) Original images and initial contours. (b) Final contours. (c) Estimated bias fields. (d) Bias-corrected images.

**Figure 3:**
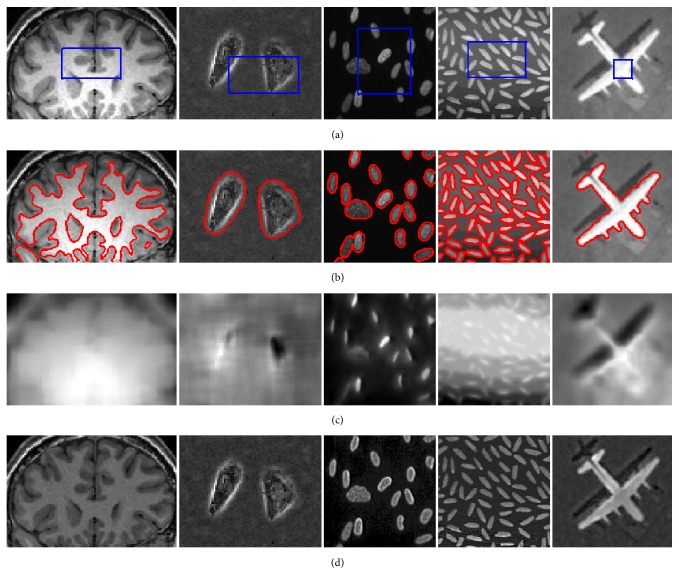
Application of our method to real images. (a) Original images and initial contours. (b) Final contours. (c) Estimated bias fields. (d) Bias-corrected images.

We firstly apply our method to segment five synthetic images, which are displayed in Figure 2(a). These images are corrupted by strong noise and intensity inhomogeneity. The final segmentation results obtained after the convergence of our algorithm are displayed in Figure 2(b). The computed bias field and the bias-corrected images are shown in Figures 2(c) and 2(d). As the local regional difference is considered, incorrect estimations of the true image in local region can be corrected in every iteration step. It can be seen from Figure 2 that the new contours gradually emerge during the evolution process. In the final segmentation results, the complete object boundaries can be effectively extracted despite the impact of intensity inhomogeneity or heavy noise. We also test our method on real images as shown in Figure 3. It reveals that the proposed model can segment multiple objectives successfully in both real images via driving the contours to desirable boundaries.

### 4.2. Segmentation of Medical Images

**Figure 4:**
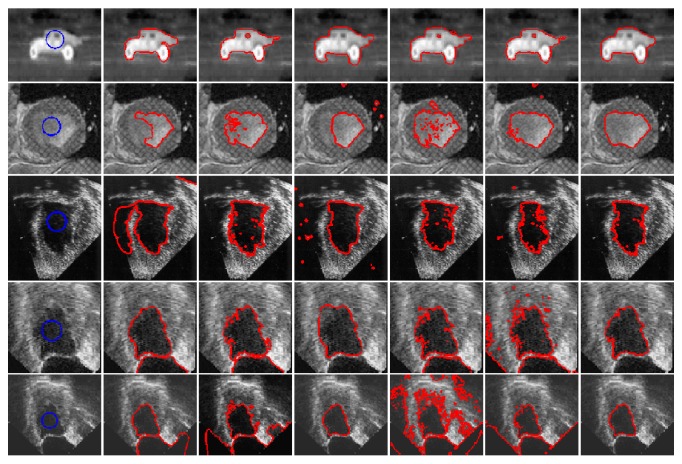
Comparison of different methods for medical images. The first column: original images and initial contours. The second row: results of RSF model. The third row: results of Li's model. The fourth row: results of LGDF model. The fifth row: results of Zhang's model. The sixth row: results of Wang's model. The last row: results of our method (Image 1: *ν* = 0.03*∗*255*∗*255; Image 2: *σ* = 5, *ν* = 0.005*∗*255*∗*255; Image 3: *σ* = 5, *ν* = 0.02*∗*255*∗*255; Image 4: *σ* = 5, *ν* = 0.01*∗*255*∗*255; Image 5: *σ* = 5, *ν* = 0.025*∗*255*∗*255).

We also evaluate the performance of our model on five medical images with obvious intensity inhomogeneity and high noise.  Figure 4 shows the segmentation results by RSF model, Li's model, LGDF model, Zhang's model, Wang's model, and the proposed model. It is obvious that the RSF model, Li's model, and Wang's method which use the Euclidean distance as a criterion of classification cannot detect the boundaries correctly. Taking more statistical characteristics into account, LGDF method and Zhang's method yield similar visual quality to our model in some images. In order to test the performance of our model, the computational time and iterations for segmentation are presented in Table 1. Compared to LGDF method and Zhang's method, the proposed model is much easier to converge. The reason is that, with the local entropy, only a simple alternating optimization is needed in every iteration step. Experiments have proven that our method has higher computing efficiency besides the accurate segmentation.

### 4.3. Segmentation of Noise Images

**Figure 5:**
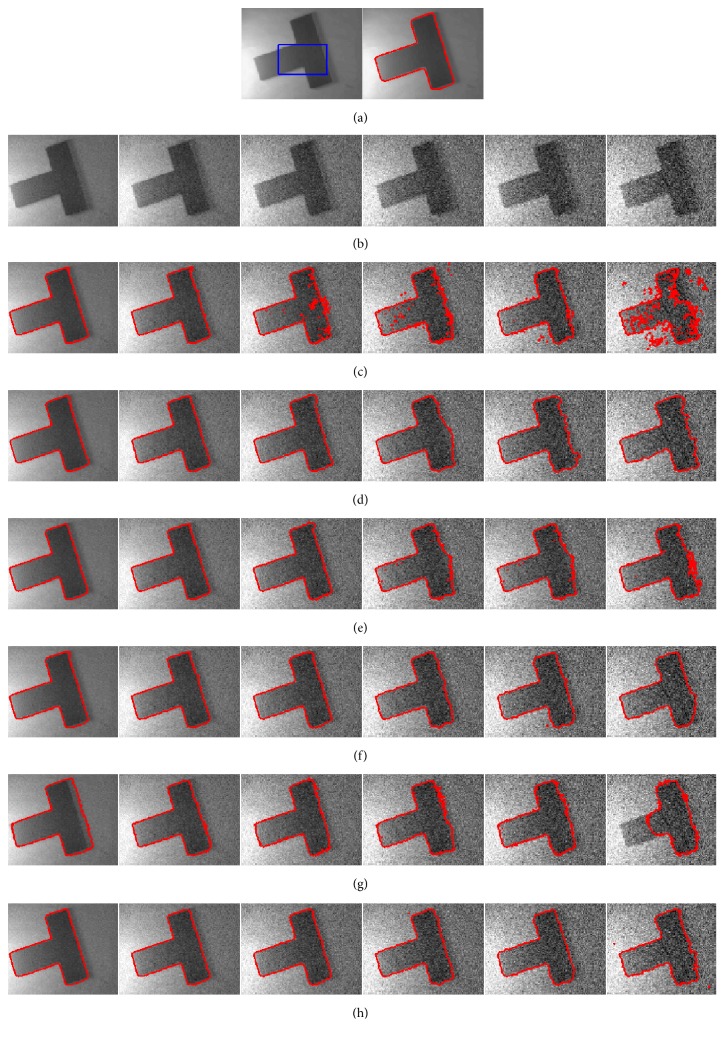
Comparison results for synthetic images polluted by various levels of Gaussian noise. (a) The original image with the ground truth. (b) shows the images with noise levels {0.05, 0.1, 0.15, 0.2, 0.25, 0.3}, respectively. (c)–(h) show the segmentation result by RSF model, Li's model, LGDF model, Zhang's model, Wang's model, and our method.

In order to evaluate the sensitivity to noise, we apply our method to the images corrupted by various levels of Gaussian noise, as shown in Figure 5.  Figure 5(a) shows the original images with the ground truth.  Figure 5(b) shows the images with Gaussian noise levels {0.05, 0.1, 0.15, 0.2, 0.25, 0.3}, respectively. We observe from Figure 5 that RSF, Li's, LGDF, and Wang's models successfully extract the object when images are corrupted by noise of lower strength, while the other two models can segment both objects from all noisy images successfully. To further illustrate the effectiveness of our method, we utilize the dice similarity coefficient (DSC) [22–24] to evaluate the performances of both models quantitatively. If *S*_1_ and *S*_2_ stand for the areas enclosed by contours obtained by the model and the manual method, respectively, then the DSC metric is defined as follows:

(24) $$\begin{array}{c} DSC=\frac{2N\left(S_{1}\cap S_{2}\right)}{N\left(S_{1}\right)+N\left(S_{2}\right)}, \end{array}$$

where *N*(∙) indicates the number of pixels in the enclosed region.

**Figure 6:**
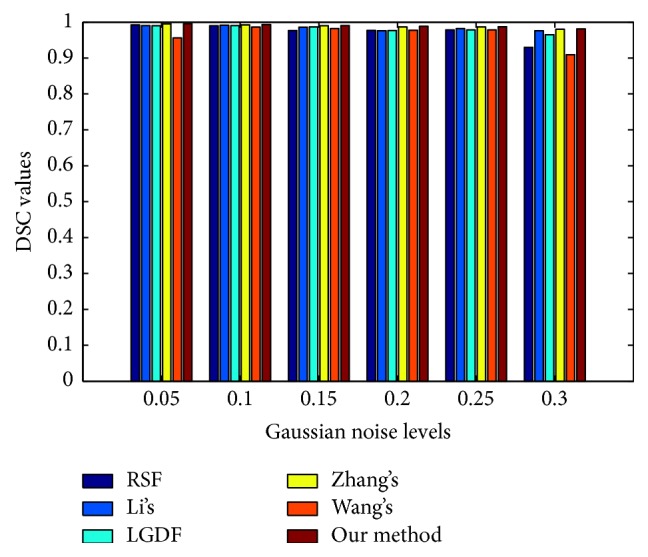
DSC values for the images corrupted by Gaussian noise in Figure 5 in the same order.

The DSC indices by applying the three compared methods are reported in Figure 6, from where it is seen that the DSC value of our model is larger than those of other methods. This demonstrates the superior performance of our method in segmentation of the images with high noise. By quantitative comparison, we can see the proposed model has good robustness to Gaussian noise.

### 4.4. Quantitative Evaluation

**Figure 7:**
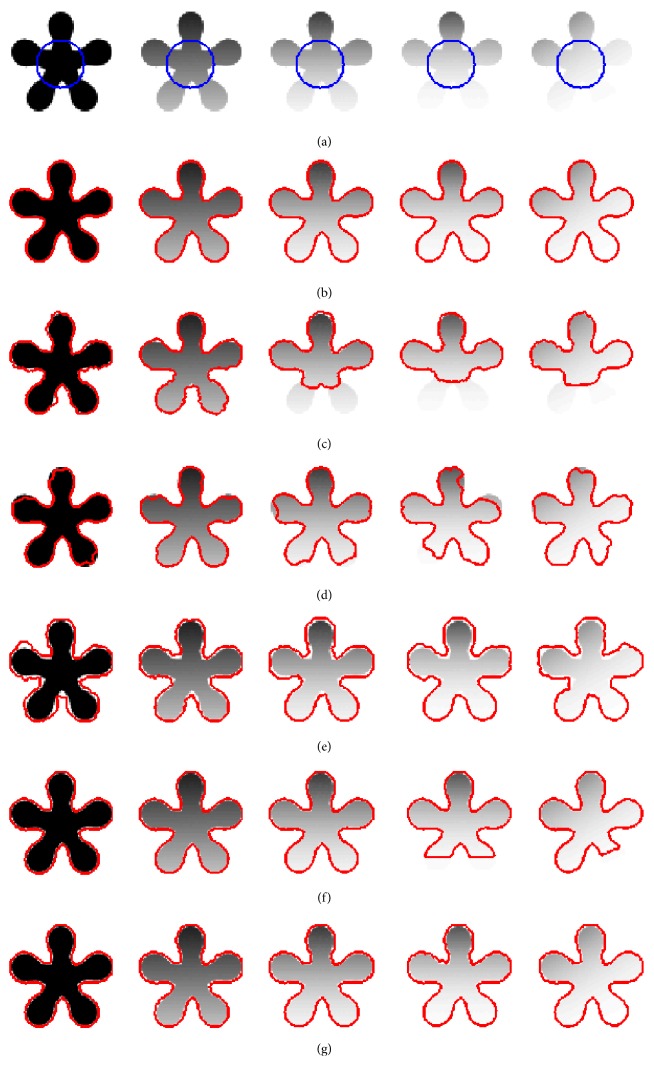
Segmentation results of five star synthetic images with different levels of intensity. (a) Original image with initial contours. (b)–(g) show the segmentation result by RSF model, Li's model, LGDF model, Zhang's model, Wang's model, and our method.

**Figure 8:**
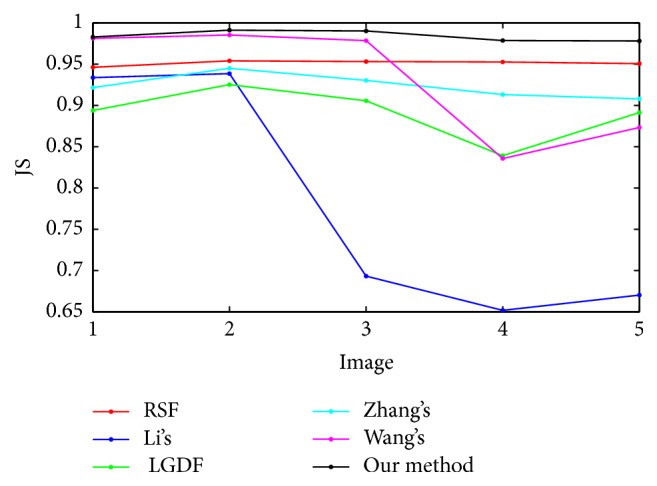
The JS values corresponding to Figure 7.

Figure 7 shows five stars images with different degree of intensity inhomogeneity.  Figure 7(a) represents original image with initial contours, whereas the segmentation results obtained by RSF method, Li's method, LGDF method, Zhang's method, Wang's method, and our method are shown from Figure 7(b) to Figure 7(g), respectively. Visual inspection clearly shows that all algorithms can segment the images precisely when intensity inhomogeneity is not strong, as shown in the first and second columns. With the increasing of intensity inhomogeneity, segmentation results of Li's method, LGDF method, and Wang's method show that they are not able to strictly find the object boundary. To further measure the quality of the extracted objects, Jaccard similarity (JS) [25, 26] is used as a quantitative measure to evaluate the segmentation results of six methods. The JS index is the ratio between two regions *S*_1_ and *S*_2_, which can be defined as JS = |*S*_1_∩*S*_2_|/|*S*_1_ ∪ *S*_2_|. The corresponding JS values for Figure 7 are shown in Figure 8. It can be seen that our method is superior in terms of accuracy than the other models even if strong intensity inhomogeneity exists. This means that our method is very robust to image intensity inhomogeneity.

**Figure 9:**
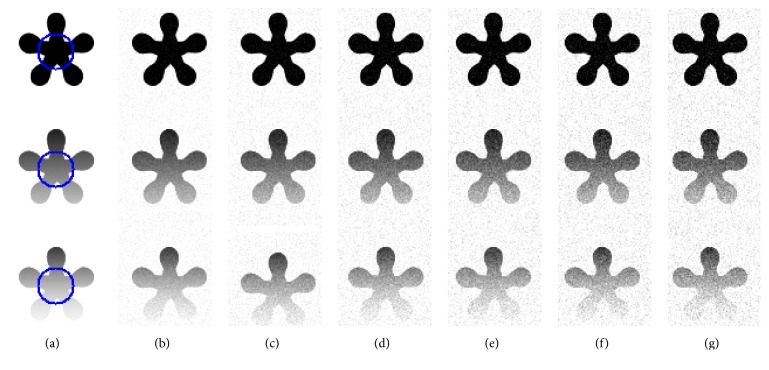
Segmentation results of stars images with different levels of Gaussian noise. (a) The original image with initial contours (the first three columns of Figure 7). (b)–(g) show the images with noise levels {0.001, 0.002, 0.003, 0.004, 0.005, 0.006}, respectively.

As shown in Figure 9, we carry out the experiments with Gaussian noise levels ranging from 0.1% to 0.6%. The JS values of these algorithms are listed in Table 2 and the best results of these algorithms are in bold. From this table, we can see that the proposed method obtained the highest JS values in spite of high level noise. This analysis indicates that our method also has good robustness toward the levels of noise.

**Figure 10:**
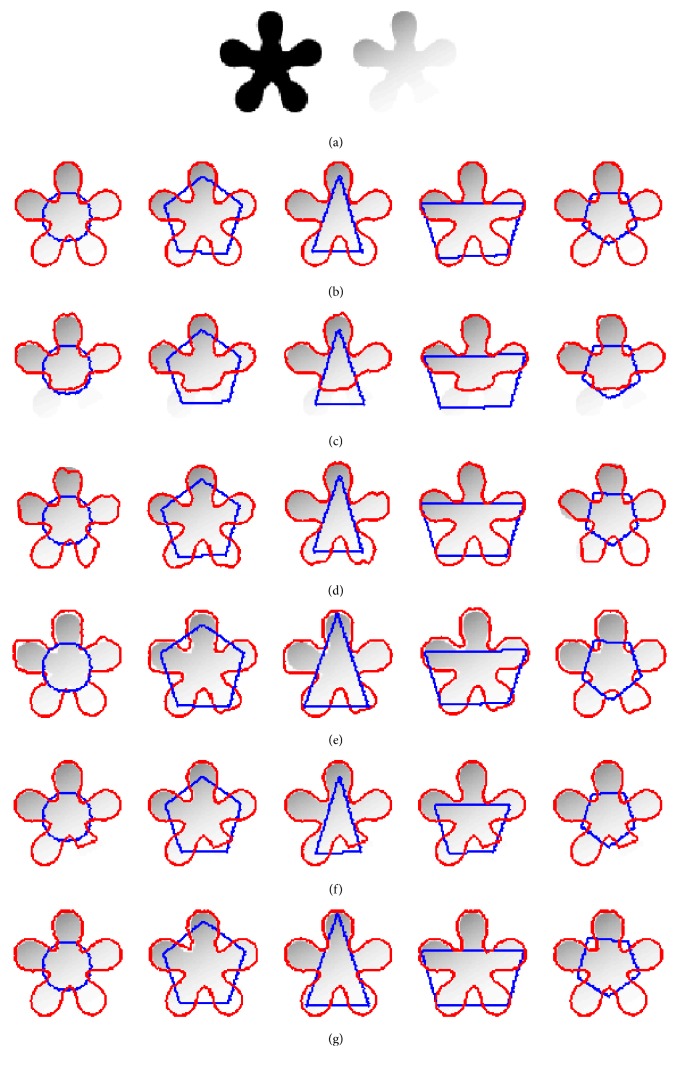
Comparison results on synthetic images with different initial contours. (a) Original image with strong intensity inhomogeneity. (b)–(g) show the segmentation result by RSF model, Li's model, LGDF model, Zhang's model, Wang's model, and our method.

**Figure 11:**
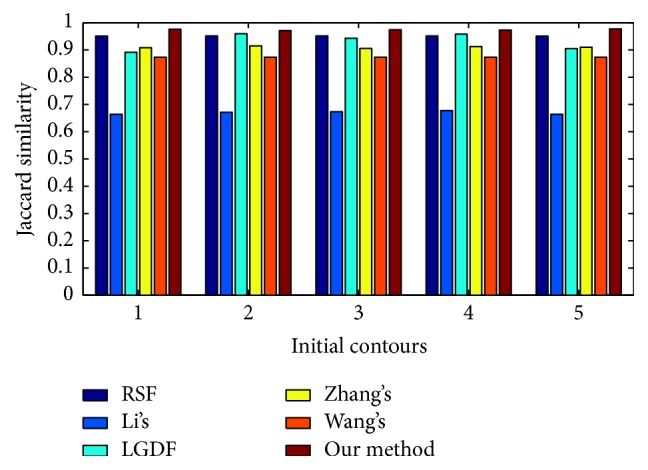
JS values for the images with different initial contours in Figure 10.

To further measure the quality of the extracted objects, we test these methods on a strong intensity inhomogeneity image with different initial contours. The results of the compared methods are reported in Figure 10. The corresponding JS values for Figure 10 are shown in Figure 11. It can be seen that our method is superior in terms of accuracy than the other models even if strong intensity inhomogeneity exists. Moreover, the JS values have few differences for different initial contours. This means that our method is very robust to initial contours.

### 4.5. Analysis for the Parameters

**Figure 12:**
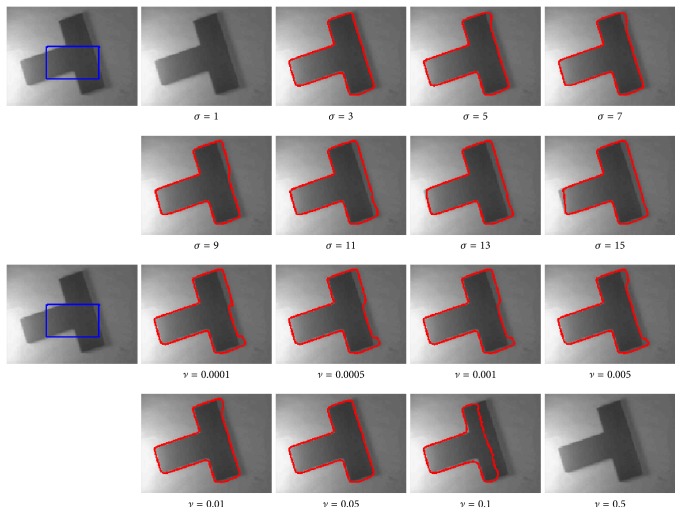
Experimental results of our model with different coefficients of length term *ν*  (*∗*255*∗*255) and scale parameter *σ*.

In this section, we simply discuss the parameters that need to be manually given to obtain appropriate segmentation results. Generally, time step Δ*t*, penalty term coefficient *μ*, binary value *c*_0_, weighting coefficients *λ*_1_ and *λ*_2_, and the positive constant of Heaviside function *ε* are relatively stable for all the experiment images. However, the parameters *σ* and *ν* seem to be sensitive. Therefore, it is necessary to discuss the relationship between the segmentation results and these parameters, fixing the other parameters and only changing one parameter each time, using the images in Figure 5. From the segmentation results shown in Figure 12, it is illustrated that the scale parameter *σ* is the standard deviation of Gaussian kernel. Increasing the value of *σ* will introduce more local image information. Hence, higher *σ* may lead to oversmoothed segmentation of the images with abundant details and textures. The regularity term coefficient *ν* can be adjusted to smooth the curves in a way that the smoothness of the curve increases when *ν* increases. On the contrary, when *ν* is too small, the results may be smooth enough and the obtained contour is sensitive to noise.

## 5. Conclusion

In this paper, we propose a novel LGDF model based on local entropy to simultaneously correct the bias field and segment the images. The local Gaussian distribution fitting term is responsible for attracting the contour toward object boundaries. By including the local entropy, our method can handle noise and intensity inhomogeneity efficiently. The experimental results on synthetic and medical images show the superiority of our method over several state-of-the-art active contour models. However, our model cannot segment images with different tissue types, such as brain MRI or tumor PET images. In the future, our model will be extended from two-phase to multiphase level set formulation, which would further enhance its capability in processing more complex medical images.

## Figures and Tables

**Table 1:** Comparison of iterations and computational time(s) for the images in Figure 4 in the same order.

Image	RSF model		Li's model		LGDF model		Zhang's model		Wang's model		Our method	
	Iterations	Time	Iterations	Time	Iterations	Time	Iterations	Time	Iterations	Time	Iterations	Time
1	70	2.11	30	16.30	150	5.86	60	4.79	200	11.26	60	3.74
2	300	24.68	90	8.29	280	8.56	150	40.73	800	28.22	100	15.13
3	200	35.41	100	28.42	120	35.84	150	42.48	500	41.06	80	13.85
4	140	8.47	50	5.82	130	19.51	80	15.18	300	6.35	40	10.78
5	25	3.84	10	2.06	200	15.84	120	21.10	400	7.11	60	11.65

**Table 2:** The JS values for images shown in Figure 9.

Image	Noise	RSF	Li's	LGDF	Zhang's	Wang's	Our model
1	0.1%	0.9457	0.937	0.9482	0.9321	0.9484	**0.9785**
	0.2%	0.9427	0.9392	0.9477	0.9435	0.9701	**0.9722**
	0.3%	0.9402	0.9333	0.9462	0.9428	0.9305	**0.9721**
	0.4%	0.9284	0.9351	0.9438	0.9341	0.9524	**0.9683**
	0.5%	0.9129	0.9394	0.9447	0.938	0.93	**0.9697**
	0.6%	0.8765	0.9363	0.9447	0.9202	0.9438	**0.9663**

2	0.1%	0.9531	0.9427	0.9633	0.9503	0.9595	**0.9786**
	0.2%	0.9517	0.945	0.9609	**0.9782**	0.9155	0.9752
	0.3%	0.9437	0.9468	0.9594	0.9628	0.8568	**0.9694**
	0.4%	0.9396	0.9414	0.9599	0.9608	0.8627	**0.9656**
	0.5%	0.8973	0.9482	0.9519	0.9546	0.9602	**0.957**
	0.6%	0.8641	0.9482	0.9488	**0.9536**	0.6934	0.9504

3	0.1%	0.9284	0.9414	0.9517	0.9588	0.9354	**0.9647**
	0.2%	0.9251	0.9453	0.9495	0.9466	0.9147	**0.9614**
	0.3%	0.9017	0.9427	0.9527	0.9478	0.8357	**0.9587**
	0.4%	0.8761	0.9218	0.9403	0.9301	0.8597	**0.9415**
	0.5%	0.8487	0.9324	0.9315	0.9354	0.921	**0.9486**
	0.6%	0.8414	0.9247	0.9201	0.9214	0.5478	**0.9354**
